# Efficacy and safety of emergent balloon aortic valvuloplasty as a rescue therapy for cardiogenic shock due to severe aortic stenosis in non-TAVI centers

**DOI:** 10.1186/s12872-025-05310-6

**Published:** 2025-11-25

**Authors:** Mayuka Masuda, Wataru Fujimoto, Masamichi Iwasaki, Kenzo Uzu, Takuma Sawa, Amane Kozuki, Ryo Nishio, Noritoshi Hiranuma, Makoto Takemoto, Koji Kuroda, Soichiro Yamashita, Junichi Imanishi, Takafumi Todoroki, Masanori Okuda, Hiromasa Otake

**Affiliations:** 1 Department of Cardiology, Hyogo Prefectural Awaji Medical Center, 1-1-137 Shioya, Sumoto, 656-0021 Hyogo Japan; 2 Division of Cardiovascular Medicine, Kobe University Graduate School of Medicine, Kobe, Japan; 3 Department of Cardiology, Konan Medical Center, Kobe, Japan; 4 Department of Cardiology, National Hospital Organization Kobe Medical Center, Kobe, Japan; 5 Department of Cardiology, Osaka Saiseikai Nakatsu Hospital, Osaka, Japan; 6 Department of Cardiology, Yodogawa Christian Hospital, Osaka, Japan; 7 Department of Cardiology, Ako City Hospital, Ako, Japan

**Keywords:** Aortic stenosis, Balloon aortic valvuloplasty, Cardiogenic shock, Retrospective study

## Abstract

**Background:**

The prognosis of aortic stenosis (AS) with cardiogenic shock remains poor, and optimal initial treatment remains unclear. Emergent balloon aortic valvuloplasty (BAV) is a treatment option for salvage and recent studies have reported that early release of valve obstruction by emergent BAV could improve prognosis. This study aimed to assess the efficacy and safety of emergent BAV for severe AS with cardiogenic shock.

**Methods:**

Among 8,230 patients hospitalized for heart failure, 7924 patients with heart failure unrelated to severe AS were excluded. Among the remaining 306 patients, 256 patients who developed cardiogenic shock due to other causes except severe AS were further excluded. Finally, a total of 41 patients with severe AS in cardiogenic shock were enrolled and divided into the emergent (underwent BAV within 6 h of admission, *n* = 9) and non-emergent (underwent BAV more than 6 h after admission, *n* = 16) groups, after excluding 16 patients who did not undergo BAV. The primary endpoints were the 30-day mortality rate and procedural complications. The secondary endpoints were days to withdrawal from the mechanical support device, days to initial rehabilitation, and clinical frailty scale (CFS) score at discharge.

**Results:**

In the emergent group, the time from admission to BAV was 3.0 ± 1.4 h, whereas BAV was performed 4.5 days (median) after admission in the non-emergent group. The 30-day mortality rate was not significantly different between the emergent and non-emergent groups (0% vs. 25%, *p* = 0.260); furthermore, there was no statistically significant difference regarding the incidence of procedural complications (0% in the emergent vs. 12.5% in the non-emergent group, *p* = 0.520). The days to withdrawal from mechanical support device and to start rehabilitation were earlier in emergent group (2.9 ± 1.2 days vs. 7.8 ± 4.6 days; *p* = 0.008, 4.2 ± 1.9 days vs. 10.8 ± 6.5 days; *p* = 0.004). The CFS score at discharge in the emergent group was maintained compared to before admission (from 3.8 ± 1.0 to 3.9 ± 1.1; *p* = 0.347), whereas worsened in the non-emergent group (from 3.8 ± 0.9 to 4.6 ± 1.2; *p* = 0.032).

**Conclusions:**

Emergent BAV for cardiogenic shock is feasible, and earlier BAV may support faster recovery and help prevent deterioration of frailty.

**Supplementary Information:**

The online version contains supplementary material available at 10.1186/s12872-025-05310-6.

## Introduction

The incidence of acute decompensated heart failure (ADHF) due to severe aortic stenosis (AS) has been increasing in the aging population [1, 2]. In particular, the prognosis of patients with AS who present with cardiogenic shock remains poor [3, 4], and the optimal initial treatment remains unclear.

Surgical aortic valve replacement (SAVR), the conventional definitive therapy for AS, is associated with a worse prognosis in patients with ADHF or cardiogenic shock than in hemodynamically stable patients [3, 4]. Although transcatheter aortic valve implantation (TAVI) has been introduced as another therapeutic option for patients at high surgical risk, previous reports have revealed that the rates of procedural complications and 30-day mortality increased fivefold in emergent TAVI for patients with cardiogenic shock compared to that in elective cases [5]. In addition, not all facilities in Japan can perform TAVI, even those specializing in cardiovascular disease. As of May 2025, there are 782 cardiovascular intervention centers certified by the Japanese Association of Cardiovascular Intervention and Therapeutics (CVIT), yet only about 250 are approved for TAVI [6, 7], highlighting a significant gap in nationwide access to TAVI.

Emergent balloon aortic valvuloplasty (BAV) is a treatment option for salvaging patients with cardiogenic shock due to severe AS [8]. In comparison to TAVI, BAV is less scrutinized and more cost-effective. As a temporary procedure, it is suitable for hemodynamically unstable patients in emergency situations. Recent studies have reported that immediate release of aortic valve obstruction by emergent BAV could improve prognosis, whereas delay in performing BAV is considered to be directly related to dire outcomes [9, 10].

This study aimed to assess the efficacy and safety of emergent BAV as a rescue therapy for cardiogenic shock due to severe AS.

## Methods

### Study design and patient population

This multicenter retrospective study conducted in six non-TAVI centers (Hyogo Prefectural Awaji Medical Center, Konan Medical Center, National Hospital Organization Kobe Medical Center, Osaka Saiseikai Nakatsu Hospital, Yodogawa Christian Hospital, and Ako City Hospital) included patients hospitalized for cardiogenic shock due to severe AS between January 2015 and July 2022. Cardiogenic shock was defined as the combination of (1) administration of catecholamines, including dobutamine and milrinone, insertion of intra-aortic balloon pump (IABP), or low cardiac index (less than 2.2 L/min/m^2^) and (2) systemic hypoperfusion identified by the combination of several parameters including altered mental status (Glasgow Coma Scale score < 15), cold/clammy skin and extremities, oliguria with urine output of less than 30 mL/h, serum lactate level higher than 2.0 mmol/L (18 mg/dL), or systolic blood pressure less than 90 mmHg. The following shock statuses related to causes other than severe AS were excluded from this study: acute coronary syndrome, tamponade, stress cardiomyopathy, pulmonary embolism, myocarditis, severe aortic regurgitation, severe mitral regurgitation/stenosis, or concomitant sepsis or severe bleeding. Furthermore, despite presenting with cardiogenic shock due to severe aortic stenosis patients who did not wish invasive treatment due to their advanced age, frailty, or comorbidities, were excluded. Participants were divided into two groups: the emergent and non-emergent group. The emergent group was defined as patients who underwent BAV within 6 h of admission. This threshold was selected based on previous studies reporting urgent or rescue BAV within 6–48 h [9–11], and to emphasize early intervention before clinical deterioration. The non-emergent group included patients who underwent BAV more than 6 h after admission.

Clinical information, including baseline patient characteristics, procedural details, and clinical outcomes, was carefully reviewed using the electronic medical records from each hospital. This study was approved by the ethics committees of all six facilities, performed according to the guidelines of the Declaration of Helsinki, and registered in the UMIN Clinical Trial Registry (UMIN 000054925) on July 10th, 2024.

### BAV procedures

All BAV procedures were performed under local anesthesia following the Japanese standards using a retrograde or antegrade approach at the discretion of each operator [12, 13]. In the retrograde arterial approach, balloon catheters were delivered through a 7–10-Fr sheath via the radial, brachial, or femoral artery. In trans-radial BAV, we used CAMEL^®^ (NIPRO, Osaka, Japan) or TRIVAL^®^ (KANEKA, Osaka, Japan) which are compatible with the 7-Fr Glidesheath Slender^®^ (Terumo, Tokyo, Japan) [14]. The antegrade transseptal approach was performed using a 12–14-Fr sheath via the femoral vein. The balloon size was primarily determined based on the diameter of the left ventricular outflow tract measured via transthoracic echocardiography. Balloon inflation typically began at approximately 2–3 ml below the target volume and was gradually increased. The success criteria for BAV were defined as at least a 50% reduction of aortic mean pressure gradient or a mean aortic pressure gradient less than 40 mmHg, measured as simultaneous left ventricular and ascending aortic pressure. For patients with low-gradient severe AS, procedural success was determined solely by the ≥ 50% reduction criterion. In every case, we attempted to use a right heart catheter before and after BAV to evaluate the hemodynamic changes both pre- and post-BAV. During the procedure, intracardiac or transthoracic echocardiography was performed to assess whether the aortic valve was properly dilated or if the aortic regurgitation had worsened (Supplemental Table 1).

### Study endpoints

The primary endpoints were the 30-day mortality rates and procedural complications, which were described according to the Valve Academic Research Consortium-3 (VARC-3) criteria [15]. The secondary endpoints were days to withdrawal from the support device, days to initial rehabilitation, and clinical frailty scale (CFS) score on discharge. The days to withdrawal from the mechanical support device, days to initial rehabilitation were calculated from the date of admission. The withdrawal from the mechanical support device was defined as the time when patients free from all mechanical support device including IABP, ventilation, and non-invasive positive pressure ventilation (NIPPV). The start of rehabilitation was defined as the time when the patient started sitting in the wheelchair. The timing of withdrawal from mechanical support device and rehabilitation initiation were at the discretion of each operator. CFS before admission was retrospectively assessed by heart team clinicians based on patients’ activities of daily living prior to the onset of acute heart failure. All assessors were routinely involved in frailty assessments as part of their daily clinical practice.

### Statistical analysis

All data are presented as mean ± standard deviation for continuous variables and as frequencies (percentages) for discrete variables. Continuous variables were analyzed using Student’s t-test and the Mann–Whitney U test according to a normal or non-normal distribution, respectively. Changes in procedural parameters before and after BAV were analyzed using paired tests: a paired t-test for normally distributed data and the Wilcoxon signed-rank test for non-normally distributed data. The chi-square or Fisher’s exact test was performed to compare the proportions of categorical variables. Statistical significance was set at *p* < 0.05. Analyses were performed using the commercially available SPSS software (version 29; SPSS Inc., Chicago, IL, USA).

### Patient and public involvement

Patients and/or the public were not involved in the design, conduct, reporting, or dissemination plans of this research.

## Results

### Baseline patient characteristics

**Fig. 1:**
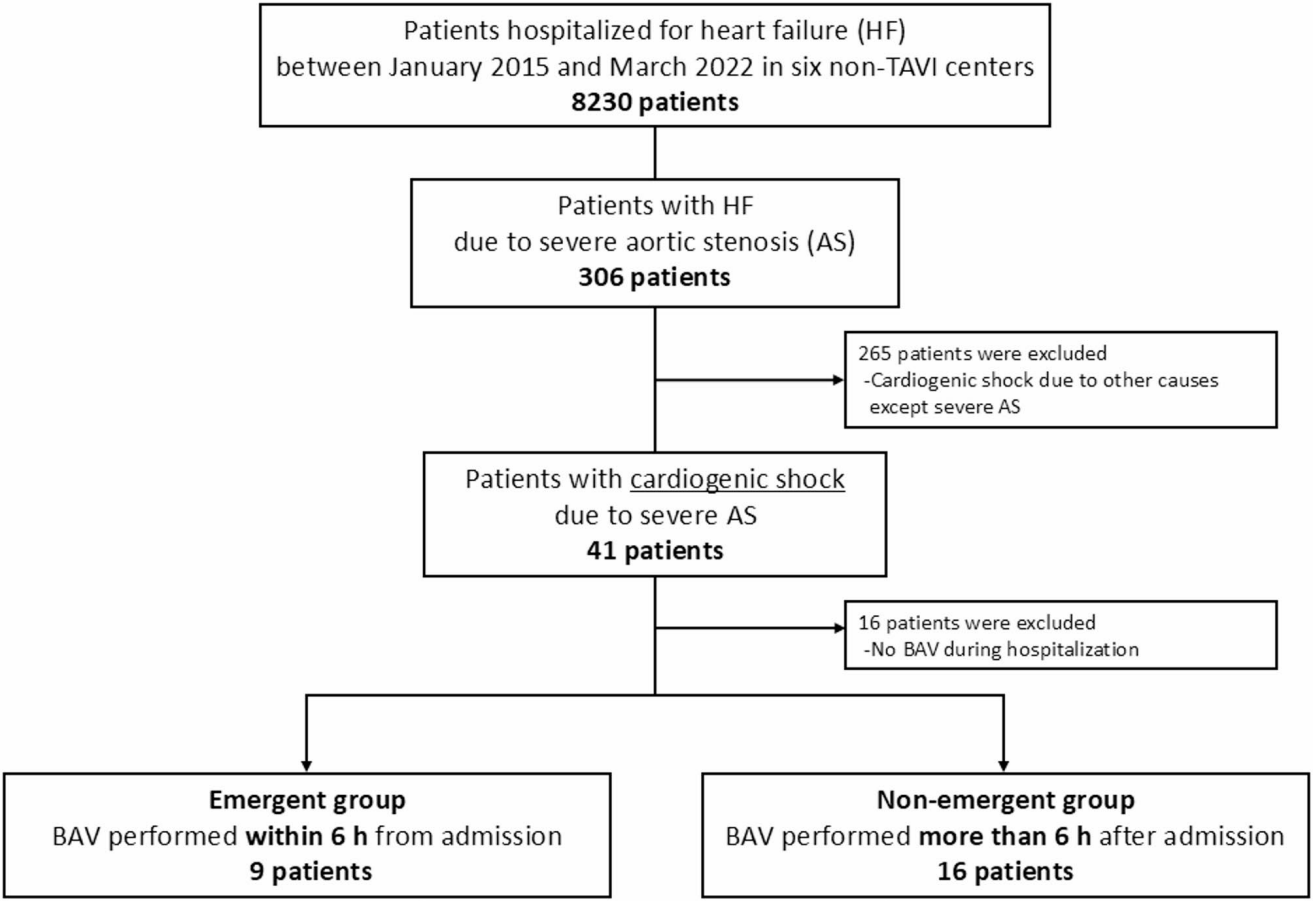
Patient selection flowchart Among 8,230 patients hospitalized for heart failure, 7924 patients with heart failure unrelated to severe AS were excluded. Of the remaining 306 patients, 256 were further excluded due to cardiogenic shock from causes other than severe AS. A total of 41 patients with severe AS and cardiogenic shock were included. After excluding 16 patients who did not undergo BAV primarily due to patient or family preference for palliative care, the remaining 25 patients were divided into the emergent group (BAV within 6 h of admission, *n* = 9) and the non-emergent group (BAV more than 6 h after admission, *n* = 16)

Among 8230 patients who were hospitalized for heart failure between January 2015 and July 2022, a total of 41 patients with cardiogenic shock due to severe AS were enrolled and divided into the emergent (*n* = 9, 22.2% male, mean age 86.7 ± 6.4 years) and non-emergent (*n* = 16, 43.8% male, mean age 88.6 ± 6.3 years) groups. No patients died while awaiting BAV. Sixteen patients (No-BAV patients) who did not undergo BAV during their hospitalization were excluded from the study (Fig. 1). The baseline characteristics showed no remarkable differences in comorbidities or the CFS score. The Society of Thoracic Surgeons (STS) score and IABP use were significantly higher in the emergent group. Additionally, in the emergent group, BAV was performed as soon as the diagnosis of cardiogenic shock due to severe AS was made; the time from admission to BAV was 3.0 ± 1.4 h. In the non-emergent group, BAV was performed 4.5 days (median) after admission (Table 1). In addition, Table 2 shows the baseline characteristics of all three groups: emergent, non-emergent, and No-BAV. The No-BAV group was included for descriptive comparison.

**Table 1:** Baseline patient characteristics

Variables	Emergent (*n* = 9)	Non-emergent (*n* = 16)	*P* value
Clinical characteristics			
Age	86.7 ± 6.4	88.6 ± 6.3	0.477
Male, n (%)	2 (22.2)	7 (43.8)	0.401
BMI (kg/m^2^)	21.1 ± 3.0	20.7 ± 6.1	0.850
NYHA class III/IV, n (%)	9 (100.0)	16 (100.0)	
Comorbidity			
Hypertension, n (%)	7 (77.8)	14 (87.5)	0.602
Diabetes, n (%)	4 (44.4)	5 (31.3)	0.671
Hemodialysis, n (%)	0 (0)	1 (6.3)	1.000
Atrial fibrillation, n (%)	3 (33.3)	4 (25.0)	0.673
Prior MI, n (%)	0 (0)	2 (12.5)	0.520
Laboratory data			
Hemoglobin (g/dL)	11.5 ± 2.7	10.5 ± 2.8	0.382
Albumin (g/dL)	3.5 ± 0.4	3.3 ± 0.6	0.370
Creatinine (mg/dL)	1.0 ± 0.3	1.6 ± 1.4	0.232
BNP (pg/mL), median [IQR]	1571.6 (1407.8–2010.1.8.1)	2062.1 (1027.5–3826.8.5.8)	0.572
Risk score			
STS score (%)	40.8 ± 13.6	19.6 ± 10.2	< 0.001
Clinical frailty scale before admission	3.8 ± 1.0	3.8 ± 0.9	0.930
Preoperative data			
LVEF (%)	38.6 ± 13.3	39.2 ± 14.0	0.907
Peak aortic velocity (m/s)	4.5 ± 0.4	4.3 ± 0.9	0.448
Mean PG (mmHg)	67.2 ± 27.7	63.4 ± 24.7	0.733
AVA (cm2)	0.4 ± 0.2	0.5 ± 0.2	0.243
AR grade ≥ 3, n (%)	0 (0)	0 (0)	
State on admission			
Catecholamine, n (%)	8 (77.8)	15 (93.8)	0.530
IABP, n (%)	4 (44.4)	2 (12.5)	0.142
Systolic BP on admission (mmHg)	96.6 ± 33.4	94.1 ± 17.3	0.843
Lactate (mg/dL)	27.9 ± 18.4	25.2 ± 27.3	0.800
NIPPV, n (%)	5 (55.6)	6 (37.5)	0.434
Ventilator, n (%)	3 (33.3)	5 (31.3)	1.000
Time to BAV from admission	3.0 ± 1.4 (hours)	4.5 (days, median)	

Qualitative data are presented as frequencies, and quantitative data are presented as means ± standard deviations or medians [interquartile ranges: IQR]

*BMI *body mass index, *NYHA *New York Heart Association, *MI *myocardial infarction, *BNP *B-type natriuretic peptide, STS Society of Thoracic Surgeons, *LVEF *left ventricular ejection fraction, *PG *pressure gradient, *AVA *aortic valve area, *AR *atrial regurgitation, *IABP *intra-aortic balloon pumping, *BP* blood pressure, *NIPPV *non-invasive positive pressure ventilation, *BAV* balloon aortic valvuloplasty

LVEF, peak aortic velocity and AR were measured using transthoracic echography, and the AVA and mean pressure gradient were calculated for simultaneous left ventricular and ascending aortic pressures

**Table 2:** Baseline characteristics of all patient groups: emergent BAV, non-emergent BAV, and no-BAV

Variables	Emergent (*n* = 9)	Non-emergent (*n* = 16)	No-BAV (*n* = 16)	*P* value
Clinical characteristics				
Age	86.7 ± 6.4	88.6 ± 6.3	88.1 ± 9.8	0.845
Male, n (%)	2 (22.2)	7 (43.8)	2 (12.5)	0.128
BMI (kg/m^2^)	21.1 ± 3.0	20.7 ± 6.1	19.7 ± 4.1	0.779
NYHA class III/IV, n (%)	9 (100.0)	16 (100.0)	16 (100)	0.264
Comorbidity				
Hypertension, n (%)	7 (77.8)	14 (87.5)	7 (43.8)	0.023
Diabetes, n (%)	4 (44.4)	5 (31.3)	2 (12.5)	0.196
Hemodialysis, n (%)	0 (0)	1 (6.3)	1 (6.3)	0.744
Atrial fibrillation, n (%)	3 (33.3)	4 (25.0)	4 (25.0)	0.883
Prior MI, n (%)	0 (0)	2 (12.5)	2 (12.5)	0.536
Laboratory data				
Hemoglobin (g/dL)	11.5 ± 2.7	10.5 ± 2.8	10.4 ± 2.8	0.596
Albumin (g/dL)	3.5 ± 0.4	3.3 ± 0.6	3.5 ± 0.6	0.665
Creatinine (mg/dL)	1.0 ± 0.3	1.6 ± 1.4	2.0 ± 1.3	0.166
BNP (pg/mL), median [IQR]	1571.6 (1407.8–2010.1.8.1)	2062.1 (1027.5–3826.8.5.8)	1523.3 (1032.5–2119.7.5.7)	0.532
Risk score				
STS score (%)	40.8 ± 13.6	19.6 ± 10.2	40.3 ± 23.1	< 0.001
CFS before admission	3.8 ± 1.0	3.8 ± 0.9	4.3 ± 1.4	0.367
Preoperative data				
LVEF (%)	38.6 ± 13.3	39.2 ± 14.0	42.1 ± 15.4	0.808
Peak aortic velocity (m/s)	4.5 ± 0.4	4.3 ± 0.9	4.2 ± 1.2	0.708
Mean PG (mmHg)	67.2 ± 27.7	63.4 ± 24.7		
AVA (cm2)	0.4 ± 0.2	0.5 ± 0.2		
AR grade ≥ 3, n (%)	0 (0)	0 (0)	0 (0)	
State on admission				
Catecholamine, n (%)	8 (77.8)	15 (93.8)	14 (87.5)	0.143
IABP, n (%)	4 (44.4)	2 (12.5)	1 (6.3)	0.042
Systolic BP on admission (mmHg)	96.6 ± 33.4	94.1 ± 17.3	89.4 ± 29.1	0.784
Lactate (mg/dL)	27.9 ± 18.4	25.2 ± 27.3	43.9 ± 33.5	0.192
NIPPV, n (%)	5 (55.6)	6 (37.5)	9 (56.3)	0.512
Ventilator, n (%)	3 (33.3)	5 (31.3)	1 (6.3)	0.150
Time to BAV from admission	3.0 ± 1.4 (hours)	4.5 (days, median)		

Qualitative data are presented as frequencies, and quantitative data are presented as means ± standard deviations or medians [interquartile ranges: IQR]

*BMI* body mass index, *NYHA* New York Heart Association, *MI* myocardial infarction, *BNP* B-type natriuretic peptide, *STS* Society of Thoracic Surgeons, *CFS* clinical frailty scale, *LVEF* left ventricular ejection fraction, *PG* pressure gradient, *AVA* aortic valve area, *AR* atrial regurgitation, *IABP* intra-aortic balloon pumping, *BP* blood pressure, *NIPPV* non-invasive positive pressure ventilation, *BAV* balloon aortic valvuloplasty

LVEF, peak aortic velocity and AR were measured using transthoracic echography, and the AVA and mean pressure gradient were calculated for simultaneous left ventricular and ascending aortic pressures

## Procedural data

Procedural data before and after BAV were shown in Table 3. In the emergent group, all cases were treated via a retrograde approach, and procedure time for BAV was shorter than in the non-emergent group (32.8 ± 8.0 min vs. 52.1 ± 16.4 min; *p* = 0.004). The BAV success rate were similar in the emergent and non-emergent group (77.8% vs. 87.5%, *p* = 0.602) and other procedural details were also comparable between the two groups. In both groups, there was a significant reduction in the mean transaortic gradient (from 67.2 ± 27.7 mmHg to 39.5 ± 22.1 mmHg; *p* = 0.049, in the emergent group, and from 63.4 ± 24.7 mmHg to 31.3 ± 15.8 mmHg; *p* < 0.001 in the non-emergent group) and an increase in the aortic valve area (from 0.4 ± 0.2 cm^2^ to 0.7 ± 0.3 cm^2^; *p* = 0.050, and from 0.5 ± 0.2 cm^2^ to 0.8 ± 0.3 cm^2^; *p* = 0.009, respectively).

**Table 3:** Procedural details and hemodynamic parameters before and after BAV

	Emergent (*n* = 9)	Non-emergent (*n* = 16)	*P* value
Retrograde approach, n (%)	9 (100)	8 (47.1)	0.022
Balloon size (mm)	19.3 ± 1.3	19.9 ± 1.6	0.349
Procedure time for BAV* (min)	32.8 ± 8.0	52.1 ± 16.4	0.004
Whole procedure time**†** (min)	88.1 ± 20.6	121.4 ± 41.1	0.041
Met study-defined success§, n (%)	7 (77.8)	14 (87.5)	0.602
Before BAV			
Mean pressure gradient (mmHg)	67.2 ± 27.7	63.4 ± 24.7	0.733
AVA (cm^2^)	0.4 ± 0.2	0.5 ± 0.2	0.243
CI (L/min/m^2^)	2.8 ± 0.6	2.5 ± 0.7	0.322
PCWP (mmHg)	25.3 ± 16.4	16.5 ± 9.5	0.158
After BAV			
Mean pressure gradient (mmHg)	39.5 ± 22.1	31.3 ± 15.8	0.327
AVA (cm^2^)	0.7 ± 0.3	0.8 ± 0.3	0.359
CI (L/min/m^2^)	3.0 ± 0.5	2.7 ± 0.6	0.334
PCWP (mmHg)	18.0 ± 7.2	13.6 ± 6.8	0.180

Quantitative data are shown as means ± standard deviations

*AVA* aortic valve area, *CI* cardiac index, *PCWP* pulmonary capillary wedge pressure

*Procedure time for BAV was defined as the time to from puncture to the completion of balloon inflating

†Whole procedure time was defined as the time from puncture to the completion, including coronary angiography, right heart catheterization, and intra-aortic balloon pumping insertion

§Procedural success was defined as at least a 50% reduction of aortic mean pressure gradient or a mean aortic pressure gradient less than 40mmHg

### Thirty-day mortality and procedural complications

The 30-day mortality rate was not significantly different between the two groups (emergent group, *n* = 0 [0%]; non-emergent group, *n* = 4 [25.0%]; *p* = 0.260; Cramér’s V = 0.327, [95% confidence interval: 0.161–0.520]). One patient in the non-emergent group died of alveolar hemorrhage, and three patients in the non-emergent group died of heart failure (*n* = 2 [13.3%]) and cardiogenic shock (*n* = 1 [6.7%]). In addition, there was no statistically significant difference between the two groups, regarding the incidence of procedural complications (emergent group, *n* = 0 [0%]; non-emergent group, *n* = 2 [12.5%]; *p* = 0.520; Cramér’s V = 0.221, [95% confidence interval: 0.115–0.387]). No vascular or access-related complications were observed and one patient experienced stroke on the day after the procedure in the non-emergent group. In addition, BAV served as a bridge to definitive therapy in several cases: 5 patients (55.6%) in the emergent group and 3 patients (18.8%) in the non-emergent group subsequently underwent TAVI or SAVR (*p* = 0.087; Table 4). Supplemental Table 2 shows detailed information of all three groups: emergent, non-emergent, and No-BAV. The No-BAV group was included for descriptive comparison.

**Table 4:** Clinical outcomes

Variables	Emergent (*n* = 9)	Non-emergent (*n* = 16)	*P* value
30-day mortality, n (%)	0 (0)	4 (25.0)	0.260
-Cardiac death, n (%)	0 (0)	2 (12.5)	
-Non cardiac death, n (%)	0 (0)	2 (12.5)	
Procedural complication, n (%)	0 (0)	2 (12.5)	0.520
-Neurologic events, n (%)	0 (0)	1 (6.3)	
-Myocardial infarction, n (%)	0 (0)	0 (0)	
-Acute kidney injury, n (%)	0 (0)	0 (0)	
-New dialysis, among patients not currently on dialysis, n (%)	0 (0)	0 (0)	
-Bleeding and transfusions, n (%)	0 (0)	1 (6.3)	
-Vascular and access-related complications, n (%)	0 (0)	0 (0)	
-New conduction disturbance and arrhythmias, n (%)	0 (0)	0 (0)	
-Post-procedural AR grade ≥ 3, n (%)	0 (0)	0 (0)	
Definitive therapy, n (%)	5 (55.6)	3 (18.8)	0.087
-Surgical aortic valve replacement, n (%)	2 (22.2)	3 (18.8)	
-Transcatheter aortic valve implantation, n (%)	3 (33.3)	0 (0)	

Qualitative data are presented with frequencies

### Days to withdrawal from mechanical support and to initial rehabilitation

**Fig. 2:**
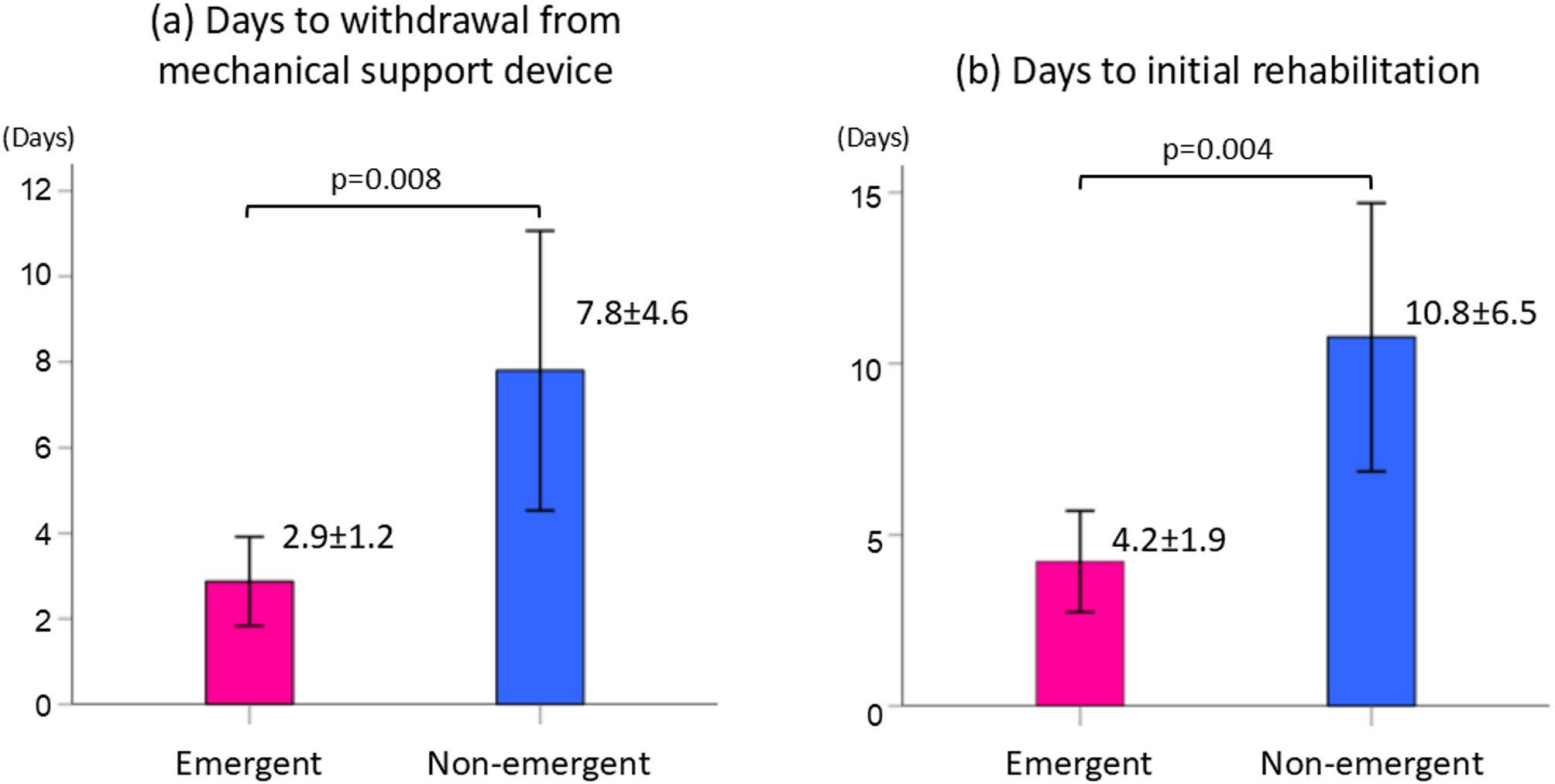
Comparison of days to withdrawal from mechanical support or to initial rehabilitation between the groups (**a**) Days to withdrawal from mechanical support, including intra-aortic balloon pump, ventilator, and non-invasive positive pressure ventilation (**b**) Days to initiation of rehabilitation in the emergent and non-emergent group Bars represent mean values with 95% confidence intervals

The days to withdrawal from mechanical support, including IABP, ventilator, and non-invasive positive pressure ventilation were 2.9 ± 1.2 days in the emergent group and 7.8 ± 4.6 days in the non-emergent group (*p* = 0.008). Moreover, the days to start rehabilitation in the two groups were 4.2 ± 1.9 days and 10.8 ± 6.5 days, respectively (*p* = 0.004) (Fig. 2).

### Comparison of the CFS score before admission and at discharge

**Fig. 3:**
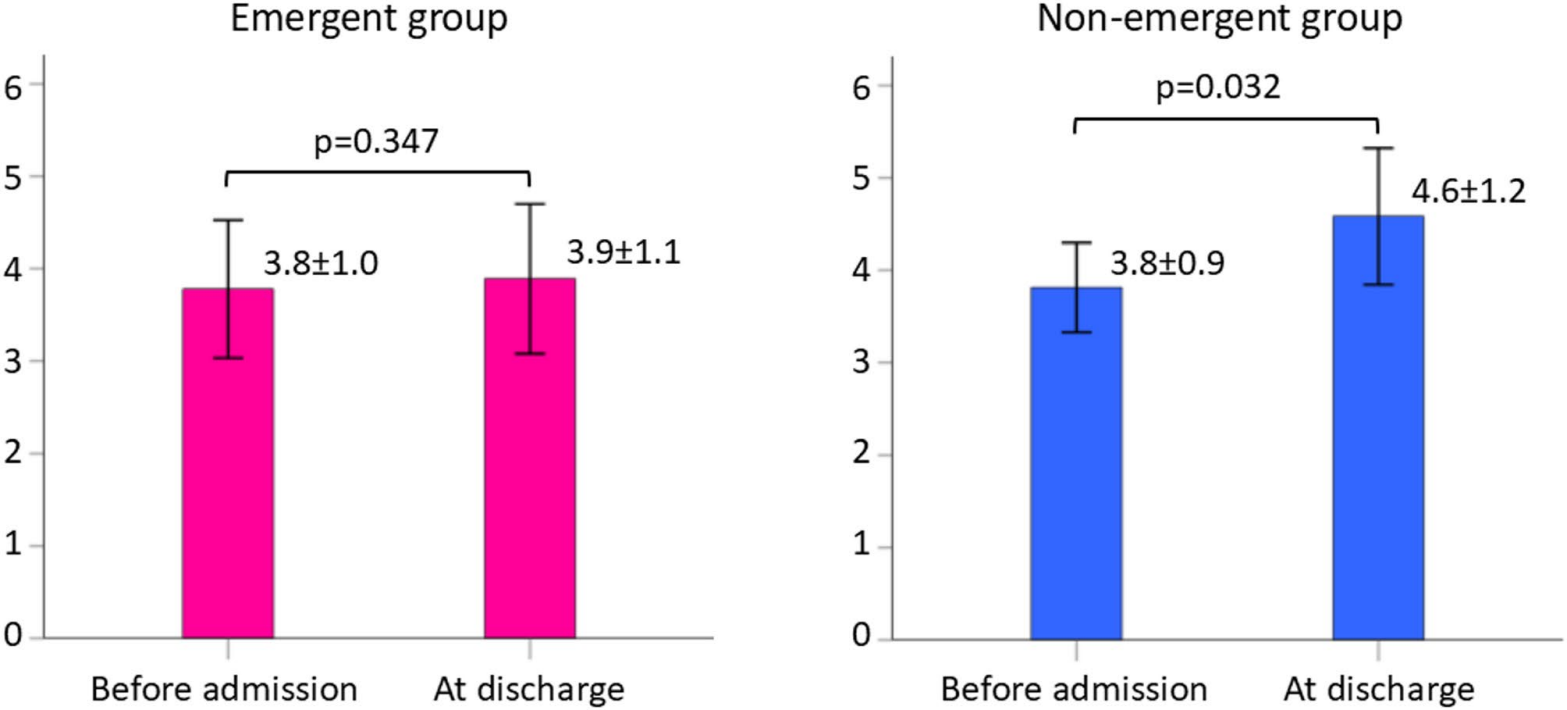
Comparison of the clinical frailty scale score before admission and at discharge. The CFS score was maintained in the emergent group but significantly worsened in the non-emergent group Bars represent mean values with 95% confidence intervals

**Fig. 4:**
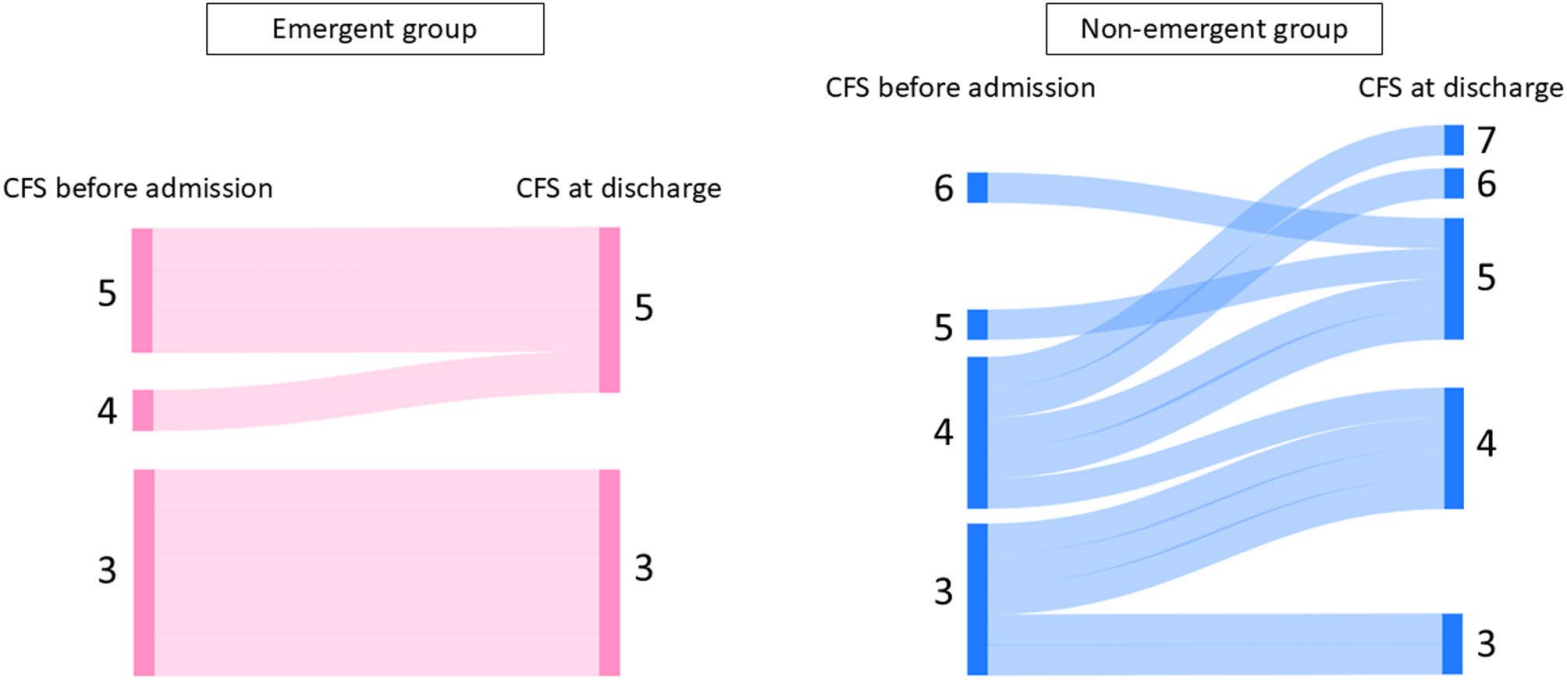
Transition of the clinical frailty scale before admission and at discharge. In the emergent group, only one patient showed worsening CFS, whereas 9 out of 16 patients in the non-emergent group experienced a decline in CFS

Comparing values before admission to values at discharge, the CFS score was maintained in the emergent group (from 3.8 ± 1.0 to 3.9 ± 1.1; *p* = 0.347) but significantly worsened in the non-emergent group (from 3.8 ± 0.9 to 4.6 ± 1.2; *p* = 0.032) (Figs. 3 and 4).

## Discussion

The current study revealed that (1) the 30-day mortality rate for patients with severe AS with cardiogenic shock undergoing BAV was 0% in the emergent group and 25.0% in the non-emergent group; (2) no intraprocedural complications were observed in this study population; (3) the emergent group required less than half the days of weaning from the ventilator and high-care units than those required by the non-emergent group; and (4) frailty did not progress in the emergent group.

### Current role of BAV

BAV was introduced as a therapeutic option by Cribier et al. in 1986 [16]. The initial reduction in the pressure gradient during the procedure was favorable; however, it did not improve the mid-term prognosis because of the high early restenosis rate. Therefore, the use of BAV decreased dramatically with the advent of TAVI in 2002. Nonetheless, BAV has recently been highlighted as a rescue therapy for hemodynamically unstable patients, even in the TAVI era [17]. National registries in Europe and the United States have disclosed an increase in BAV cases [17, 18], of which BAV for salvage accounts for approximately 10% of cases [17, 19]. The observed increase in the number of BAV cases is considered to be primarily attributable to the expanding use of BAV as a “bridge therapy” to definitive treatments (SAVR or TAVI) for critically ill patients. On the other hand, it is also implied that there remains a certain necessity of emergent BAV as a salvage therapy in real-world clinical practice.

### Outcomes of emergent BAV in patients with cardiogenic shock

In 2018, Debry et al. assessed the outcomes of BAV as a rescue therapy in patients with cardiogenic shock due to severe AS and revealed that the 30-day mortality rate was 47% [10]. Eugene et al. also reported that the rate of early death was 30% for cardiogenic shock or refractory pulmonary edema [11]. These previous reports have again depicted the poor prognosis of patients with cardiogenic shock due to severe AS; however, they also suggest that early intervention toward aortic valve obstruction will improve prognosis. Early direct intervention (within 48 h) for aortic valve obstruction can help prevent the need for high doses of catecholamines, which would decrease the risk of multi-organ failure [10]. In this study, although not statistically significant, the 30-day mortality rate in the emergent group (0%) was lower than that in the non-emergent group (25.0%), suggesting a favorable trend toward improved early outcomes with earlier intervention. However, due to the limited sample size, the difference did not reach statistical significance (*p* = 0.260). On the other hand, the effect size, represented by Cramér’s V (Phi coefficient), was calculated as 0.327, indicating a moderate effect. Moreover, post hoc power analysis showed a statistical power of only 37.2%, suggesting that the lack of statistical significance may be attributed to insufficient power. Furthermore, there is no evidence to determine how quickly treatment should be administered to be effective, but it was revealed that the sooner pulmonary edema is relieved, the better the prognosis [20]. This may explain why the emergent group, treated approximately 3.0 ± 1.4 h after admission, had better outcomes than those of patients in the non-emergent group and previously reported cases of AS with cardiogenic shock. Although statistical significance was not achieved, reducing the duration of cardiogenic shock until the release of valve obstruction may be a key factor in salvaging patients in the acute phase.

We also revealed that immediate BAV can promote withdrawal from mechanical support, facilitate rehabilitation, and prevent the deterioration of the CFS score. Being bedridden for a long duration has been known to lead to a reduction in muscle strength by 1% per day, even for a healthy adult [21]; therefore, it is important to shorten the duration in which older patients are bedridden as much as possible due to their already reduced muscle strength [22]. Moreover, the CFS score at discharge affects the prognosis of patients with heart failure [23]. When it comes to bridging TAVI, the preprocedural CFS score is reported to be an independent prognostic factor after undergoing TAVI [24]; thus, maintaining the CFS score in older patients during hospitalization is an inevitable issue for clinicians. As illustrated in Fig. 4, the emergent BAV group showed a trend toward better preservation of frailty status compared to the non-emergent group. These findings support the potential advantages of emergent BAV in preventing worsening frailty and promoting a smooth bridge to subsequent definitive therapy in critically ill older patients. In future studies, it will be essential to perform a robust multivariable assessment to determine the extent to which CFS values or the timing of BAV influenced eligibility for definitive therapy.

### Comparison of safety between emergent and non-emergent BAV

Emergent procedures for hemodynamically unstable patients are high-pressure situations for operators and are generally thought to increase the risk of intraprocedural complications; however, our study showed no significant difference in periprocedural complication rates. Previous reports have also shown lower intraprocedural complication rates in emergent BAV than in emergent TAVI [25]. While the treatment targets severe cases with poor prognosis, performing BAV in an emergency setting was not considered to be directly related to adverse events. Additionally, all institutions included in this study were primary hospitals in their respective regions but did not have approval to perform TAVI. As for BAV, a volume-outcome relationship was not observed in the nationwide registry data in Japan [13]. BAV may be advantageous in emergency situations as an easy-to-use procedure. Notably, in our cohort, no periprocedural complications or early deaths were observed among patients undergoing emergent BAV, suggesting a favorable safety profile despite the critical condition of these patients. While the limited sample size precludes definitive conclusions, these findings raise the hypothesis that emergent BAV may provide both a safe and practical bridge to stabilization in non-TAVI centers. These observations, if explored in larger cohorts, could help generate hypotheses regarding the potential benefits of early intervention, particularly in terms of reducing early mortality and preserving frailty, for future prospective studies.

### Emergent TAVI vs. Emergent BAV

Recently, the indications for TAVI have been expanding towards critically ill patients; however, the feasibility and safety of emergent TAVI for patients with cardiogenic shock remains controversial. Frerker et al. reported a 30-day mortality rate of 33.3% in emergent TAVI for cardiogenic shock, which was significantly higher than that in electively treated patients (7.7%; *p* < 0.0001) [26]. The registry in 2020 revealed that emergent TAVI for cardiogenic shock was associated with higher 30-day mortality (19.1% vs. 4.9%, *p* < 0.001) and complication rates compared to those in high-risk patients without cardiogenic shock [5].

Very few studies have directly compared emergent BAV and TAVI in cardiogenic shock. In 2018, the multicenter retrospective study investigating the early outcomes of emergent TAVI versus emergent BAV followed by elective TAVI showed the similar rates of cardiovascular mortality after 30 days in the emergent TAVI and BAV groups (23.8% vs. 33.0%, *p* = 0.40). In contrast, significant major vascular complications (*p* = 0.01) and stroke (*p* = 0.01) have been observed after emergent TAVI [27]. Moreover, the latest study evaluating the impact of emergent BAV or TAVI on outcomes in patients with cardiogenic shock revealed that BAV was associated with a short-term mortality benefit, including statistically similar in-hospital mortality rates to those for TAVR and surgical AVR [25]. However, the definition of emergent status varies depending on previous papers, which might request the careful interpretation.

Emergent TAVI has several limitations. First, the precise prosthesis selection may be challenging. The most common post-procedural complication is paravalvular leakage (PVL), and even mild PVL is associated with increased late mortality [28]. Compared to multidetector computed tomography (MDCT), two-dimensional transesophageal echocardiography is associated with a significantly higher incidence of more than moderate PVL after TAVI [29, 30]. However, in emergency situations, aortic annulus can normally be measured using echocardiography, which may increase the incidence of PVL. Second, emergent BAV is not constrained by institutional capabilities, whereas emergent TAVI may be performed in specialized centers.

BAV can confer only transient hemodynamic improvement, which is inherently different to TAVI. Of note, the benefit of emergent BAV lies in its temporary therapeutic effect. First, emergent BAV may be an especially useful strategy if the cause of cardiogenic shock is uncertain. Definitive therapies should be offered if patients respond favorably and if severe AS has been proven to be the cause of cardiogenic shock. Second, BAV may allow for better selection of patients who would benefit from definitive therapies after clinical and hemodynamic stabilization. In acute phase, it may be challenging to ascertain whether patients with shock are optimal candidates for definitive therapies, including TAVI or SAVR, in terms of frailty and comorbidities. Although this study was conducted in Japanese non-TAVI centers, the findings may be applicable to similar healthcare environments both within and beyond Japan, particularly in regions where emergent TAVI is not readily available. By highlighting the potential role of emergent BAV in such real-world settings, this study emphasizes the clinical relevance of emergent BAV in healthcare systems lacking immediate access to definitive therapies.

### Limitations

The results of our study should be interpreted in the context of some limitations. First, although this was a multicenter retrospective study, the small sample size inherently limits the statistical power. Accordingly, the results of this study should be interpreted solely as exploratory and hypothesis-generating, serving as a foundation for future investigations rather than providing confirmatory evidence. To ensure statistical robustness of our findings, larger multicenter studies or registry-based investigations are warranted. Second, because of the retrospective design, there was no uniformity in the detailed BAV procedures. This procedural heterogeneity may limit reproducibility and warrants caution in interpreting the results. To more accurately evaluate the clinical utility of early intervention, standardized procedural criteria will be required. Third, considering the inherent limitations of the retrospective design, potential selection bias and confounding cannot be excluded. The feasibility of emergent BAV were determined at the discretion of each operator; thus, treatment indication itself may have introduced confounding. Notably, patients in the emergent group tended to be more critically ill (e.g., higher STS scores and more frequent IABP use), suggesting that early intervention was often chosen for those who could not wait, while delayed cases may have initially appeared stable but later deteriorated. This complexity should be considered when interpreting the comparative outcomes. Moreover, the timing of BAV was determined by each operator based on individual clinical judgment rather than a standardized protocol, similarly introducing potential confounding. This decision may have been influenced by both clinical and non-clinical factors, which could not be fully accounted for. Although no patients died while awaiting BAV in this study, the possibility of pre-procedural mortality should be considered as a potential source of bias. Therefore, to minimize potential bias and rigorously evaluate the benefits of emergent intervention, future studies should be conducted under standardized, prospective protocols. Fourth, due to the small sample size and limited number of events, multivariable adjustment or propensity score matching to adjust for imbalances in baseline characteristics and confounders was not feasible without risking model overfitting and instability. Therefore, all observed effects of this study should be interpreted with caution, and adjusted analyses in large-scale cohorts or well-designed prospective studies will be indispensable in the future to confirm these findings. Fifth, sixteen patients did not undergo BAV, primarily due to their own or their families’ preference for palliative care. Although their baseline characteristics were comparable to those of the BAV group (Table 2), advanced age and serious comorbidities frequently influenced the decision to pursue palliative rather than invasive treatment. Consequently, the decision was not solely based on objective clinical severity but was also shaped by personal, social, and contextual factors, which may have introduced potential selection bias. Sixth, although we assessed efficacy of emergent BAV as rescue therapy using CFS, it is inherently semiquantitative and subjective in nature. Accordingly, it is susceptible to both recall and interobserver bias. To minimize subjectivity, standardized frailty assessment tools, together with assessor training and blinding, should be implemented, and inter- and intra-rater agreement should be prospectively quantified in future studies. Finally, cardiogenic shock was diagnosed retrospectively using electronic medical records, which may have led to the underreporting of cases in which the diagnosis was not accurately recorded.

In light of these limitations, the findings of this study should be interpreted with caution. The retrospective design, small sample size, procedural heterogeneity, and potential for selection bias collectively limit the internal validity and generalizability of the results. Furthermore, the inability to perform robust statistical adjustments and the use of subjective assessment tools introduce additional methodological constraints. Despite these limitations, the study provides valuable preliminary insights into the clinical utility of emergent BAV for patients with cardiogenic shock, warranting further investigation through prospective, larger-scale studies.

## Conclusion

Emergent BAV for cardiogenic shock may serve as a feasible and effective rescue therapy. Earlier BAV could contribute to immediate recovery, help prevent frailty, and provide a smooth bridge to subsequent treatment in patients with cardiogenic shock due to severe AS.

## Supplementary Information

Supplementary Material 1.

Supplementary Material 2.

Supplementary Material 3.

## Data Availability

The datasets generated and/or analyzed during the current study are available from the corresponding author on reasonable request. Requests for data access should be directed to Dr. Masamichi Iwasaki (e mail: iwa_michi@awajimc.jp).
