# Supplementary material for: Efficacy and safety of emergent balloon aortic valvuloplasty as a rescue therapy for cardiogenic shock due to severe aortic stenosis in non-TAVI centers
Source: BMC Cardiovasc Disord. 2025 Nov 25;25:836. doi: 10.1186/s12872-025-05310-6 (PMC12648894; doi:10.1186/s12872-025-05310-6)
Supplement: Supplementary file 1 — Supplementary Material 1. [file 12872_2025_5310_MOESM1_ESM.docx]

**Supplemental table 1. Individual procedural data of all 25 patients who underwent BAV**

|  | | **Time to BAV from admission** | **Approach** | **Puncture site** | **Sheath size (Fr)** | **Balloon size (mm)** |
| --- | --- | --- | --- | --- | --- | --- |
| **Emergent group** | |  |  |  |  |  |
| Case | 1 | 323 (min) | Retrograde | FA | 8 | 20 |
|  | 2 | 289 (min) | Retrograde | FA | 8 | 20 |
|  | 3 | 189 (min) | Retrograde | FA | 10 | 18 |
|  | 4 | 62 (min) | Retrograde | FA | 9 | 19 |
|  | 5 | 99 (min) | Retrograde | FA | 10 | 22 |
|  | 6 | 111 (min) | Retrograde | BA | 7 | 20 |
|  | 7 | 157 (min) | Retrograde | RA | 7 | 18 |
|  | 8 | 190 (min) | Retrograde | FA | 8 | 18 |
|  | 9 | 204 (min) | Retrograde | RA | 7 | 19 |
| **Non-emergent group** | |  |  |  |  |  |
| Case | 1 | 0 (days) | Retrograde | FA | 8 | 18 |
|  | 2 | 5 (days) | Retrograde | FA | 10 | 18 |
|  | 3 | 26 (days) | Retrograde | RA | 7 | 20 |
|  | 4 | 14 (days) | Antegrade | FV | 12 | 20 |
|  | 5 | 7 (days) | Retrograde | RA | 7 | 18 |
|  | 6 | 4 (days) | Antegrade | FV | 14 | 22 |
|  | 7 | 3 (days) | Antegrade | FV | 14 | 22 |
|  | 8 | 6 (days) | Antegrade | FV | 14 | 22 |
|  | 9 | 5 (days) | Retrograde | FA | 10 | 20 |
|  | 10 | 1 (day) | Antegrade | FV | 14 | 22 |
|  | 11 | 10 (days) | Antegrade | FV | 12 | 21 |
|  | 12 | 2 (days) | Retrograde | FA | 8 | 20 |
|  | 13 | 12 (days) | Antegrade | FV | 12 | 19 |
|  | 14 | 1 (day) | Antegrade | FV | 14 | 21 |
|  | 15 | 3 (days) | Retrograde | FA | 10 | 18 |
|  | 16 | 3 (days) | Retrograde | FA | 7 | 18 |

FA, femoral artery; BA, brachial artery; RA, radial artery; FV, femoral vein
