# Supplementary material for: Efficacy and safety of emergent balloon aortic valvuloplasty as a rescue therapy for cardiogenic shock due to severe aortic stenosis in non-TAVI centers
Source: BMC Cardiovasc Disord. 2025 Nov 25;25:836. doi: 10.1186/s12872-025-05310-6 (PMC12648894; doi:10.1186/s12872-025-05310-6)
Supplement: Supplementary file 2 — Supplementary Material 2. [file 12872_2025_5310_MOESM2_ESM.docx]

**Supplemental table 2. Individual baseline patient characteristics data of all three groups: emergent, non-emergent, and no-BAV**

|  |  | Age | Sex | NYHA | STS score (%) | CFS before admission | CFS on discharge | Days to withdrawal  from the support device (days) | Days to initial  rehabilitation (days) | 30-day mortality | Definitive therapy |
| --- | --- | --- | --- | --- | --- | --- | --- | --- | --- | --- | --- |
| **Emergent group** | |  |  |  |  |  |  |  |  |  |  |
| Case | 1 | 73 | Female | 4 | 23.0 | 3 | 3 | 4 | 5 | Alive | NA |
|  | 2 | 89 | Male | 4 | 46.6 | 5 | 5 | 6 | 6 | Alive | SAVR |
|  | 3 | 90 | Female | 4 | 36.8 | 4 | 5 | 3 | 3 | Alive | NA |
|  | 4 | 86 | Female | 4 | 27.6 | 3 | 3 | 5 | 5 | Alive | TAVI |
|  | 5 | 93 | Female | 4 | 66.0 | 3 | 3 | 3 | 3 | Alive | NA |
|  | 6 | 88 | Female | 4 | 45.7 | 5 | 5 | 2 | 2 | Alive | TAVI |
|  | 7 | 89 | Female | 4 | 41.5 | 5 | 5 | 3 | 3 | Alive | NA |
|  | 8 | 92 | Female | 4 | 51.7 | 3 | 3 | 3 | 3 | Alive | TAVI |
|  | 9 | 80 | Male | 4 | 28.1 | 3 | 3 | 8 | 8 | Alive | SAVR |
| **Non-emergent group** | |  |  |  |  |  |  |  |  |  |  |
| Case | 1 | 77 | Female | 4 | 34.6 | 3 | NA | NA | NA | Non-CV death | NA |
|  | 2 | 92 | Female | 4 | 15.3 | 3 | 4 | 7 | 9 | Alive | NA |
|  | 3 | 95 | Female | 4 | 11.5 | 3 | 4 | NA | 17 | Alive | NA |
|  | 4 | 91 | Female | 4 | 21.3 | 4 | 7 | NA | 27 | Alive | NA |
|  | 5 | 93 | Female | 4 | 13.7 | 5 | NA | NA | NA | CV death | NA |
|  | 6 | 92 | Male | 4 | 21.9 | 4 | 6 | 5 | 6 | Alive | NA |
|  | 7 | 78 | Male | 4 | 7.7 | 3 | 3 | 5 | 6 | Alive | SAVR |
|  | 8 | 86 | Female | 4 | 20.5 | 4 | 5 | 15 | 15 | Alive | NA |
|  | 9 | 92 | Male | 3 | 7.9 | 6 | 5 | 5 | 15 | Alive | NA |
|  | 10 | 82 | Male | 4 | 16.7 | 3 | NA | 6 | 7 | CV death | SAVR |
|  | 11 | 85 | Male | 4 | 16.0 | 3 | 3 | 9 | 10 | Alive | SAVR |
|  | 12 | 87 | Male | 3 | 7.7 | 4 | 5 | 4 | 2 | Alive | NA |
|  | 13 | 101 | Female | 4 | 24.3 | 4 | 4 | 5 | 11 | Alive | NA |
|  | 14 | 88 | Female | 4 | 23.6 | 5 | 5 | 17 | 9 | Alive | NA |
|  | 15 | 86 | Female | 3 | 25.8 | 3 | 4 | NA | 6 | Alive | NA |
|  | 16 | 92 | Male | 4 | 45.8 | 4 | NA | NA | NA | Non-CV death | NA |
| **No-BAV group** | |  |  |  |  |  |  |  |  |  |  |
| Case | 1 | 68 | Female | 4 | 4.4 | 3 | 3 | 9 | 10 | Alive | SAVR |
|  | 2 | 92 | Female | 4 | 11.6 | 5 | 5 | 6 | 12 | Alive | NA |
|  | 3 | 69 | Female | 4 | NA | 3 | 3 | 3 | 5 | Alive | SAVR |
|  | 4 | 92 | Female | 4 | 47.4 | 7 | NA | NA | NA | CV death | NA |
|  | 5 | 93 | Female | 4 | 18.7 | 5 | NA | NA | NA | CV death | NA |
|  | 6 | 88 | Female | 4 | 23.3 | 3 | NA | NA | NA | CV death | NA |
|  | 7 | 94 | Female | 4 | 19.5 | 3 | NA | NA | NA | CV death | NA |
|  | 8 | 91 | Female | 4 | 38.0 | 4 | NA | NA | NA | CV death | NA |
|  | 9 | 104 | Female | 4 | 53.8 | 7 | 7 | 8 | 8 | Alive | NA |
|  | 10 | 82 | Female | 4 | 58.2 | 4 | NA | NA | NA | Non-CV death | NA |
|  | 11 | 94 | Female | 4 | 41.5 | 4 | NA | 3 | NA | CV death | NA |
|  | 12 | 95 | Female | 4 | 67.9 | 4 | 4 | 2 | 3 | Alive | TAVI |
|  | 13 | 93 | Male | 4 | 82.5 | 6 | NA | NA | NA | CV death | NA |
|  | 14 | 92 | Female | 3 | 62.6 | 4 | NA | NA | NA | CV death | NA |
|  | 15 | 75 | Male | 4 | 34.3 | 4 | NA | NA | NA | CV death | NA |
|  | 16 | 87 | Female | 4 | NA | 3 | NA | NA | NA | CV death | NA |

BAV, balloon aortic valvuloplasty; CFS, clinical frailty scale; CV, cardiovascular NA, not applicable; NYHA, New York Heart Association; SAVR, surgical aortic valve replacement; STS, Society of Thoracic Surgeons; TAVI, transcatheter aortic valve implantation.
