# Supplementary figures and images for: Efficacy and safety of emergent balloon aortic valvuloplasty as a rescue therapy for cardiogenic shock due to severe aortic stenosis in non-TAVI centers
Source: BMC Cardiovasc Disord. 2025 Nov 25;25:836. doi: 10.1186/s12872-025-05310-6 (PMC12648894; doi:10.1186/s12872-025-05310-6)

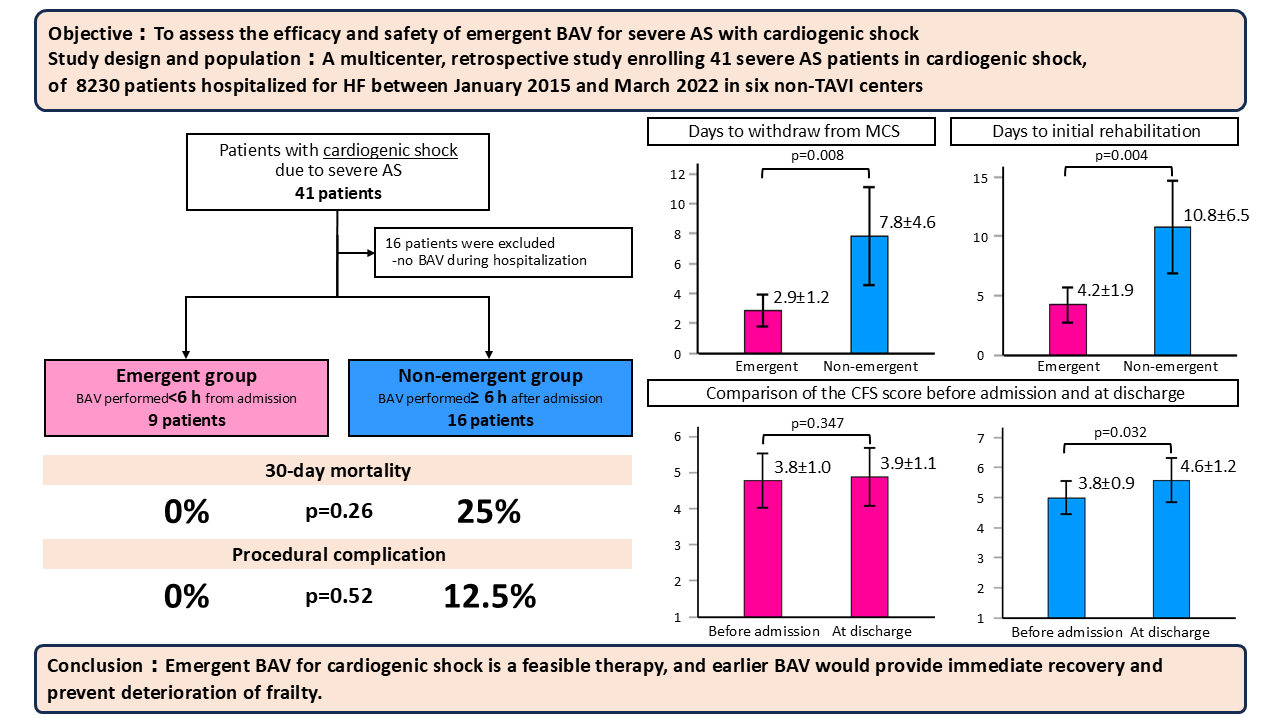

Supplement: Supplementary file 3 — Supplementary Material 3. [file 12872_2025_5310_MOESM3_ESM.tif]
